# Network Signatures of Survival in Glioblastoma Multiforme

**DOI:** 10.1371/journal.pcbi.1003237

**Published:** 2013-09-19

**Authors:** Vishal N. Patel, Giridharan Gokulrangan, Salim A. Chowdhury, Yanwen Chen, Andrew E. Sloan, Mehmet Koyutürk, Jill Barnholtz-Sloan, Mark R. Chance

**Affiliations:** 1 Center for Proteomics and Bioinformatics, Case Western Reserve University, Cleveland, Ohio, United States of America; 2 School of Computer Science, Carnegie Mellon University, Pittsburgh, Pennsylvania, United States of America; 3 Case Comprehensive Cancer Center, Case Western Reserve University, Cleveland, Ohio, United States of America; 4 Brain Tumor & Neuro-Oncology Center, University Hospital-Case Medical Center, Cleveland, Ohio, United States of America; 5 Department of Electrical Engineering & Computer Science, Case Western Reserve University, Cleveland, Ohio, United States of America; Tufts University, United States of America

## Abstract

To determine a molecular basis for prognostic differences in glioblastoma multiforme (GBM), we employed a combinatorial network analysis framework to exhaustively search for molecular patterns in protein-protein interaction (PPI) networks. We identified a dysregulated molecular signature distinguishing short-term (survival<225 days) from long-term (survival>635 days) survivors of GBM using whole genome expression data from The Cancer Genome Atlas (TCGA). A 50-gene subnetwork signature achieved 80% prediction accuracy when tested against an independent gene expression dataset. Functional annotations for the subnetwork signature included “protein kinase cascade,” “IκB kinase/NFκB cascade,” and “regulation of programmed cell death” – all of which were not significant in signatures of existing subtypes. Finally, we used label-free proteomics to examine how our subnetwork signature predicted protein level expression differences in an independent GBM cohort of 16 patients. We found that the genes discovered using network biology had a higher probability of dysregulated protein expression than either genes exhibiting individual differential expression or genes derived from known GBM subtypes. In particular, the long-term survivor subtype was characterized by increased protein expression of DNM1 and MAPK1 and decreased expression of HSPA9, PSMD3, and CANX. Overall, we demonstrate that the combinatorial analysis of gene expression data constrained by PPIs outlines an approach for the discovery of robust and translatable molecular signatures in GBM.

## Author Summary

Glioblastoma multiforme (GBM) is the most common and aggressive brain tumor in adults, and, while the median survival time for treated patients is approximately one year, subgroups of patients respond differently to the same treatments, with some patients showing little improvement and other patients living far longer than expected. These differences in treatment response indicate that the tumors may show molecular differences that we can harness to tailor cancer therapy. To this end, we sought to identify biomarkers of patient survival in GBM. To improve the applicability of our molecular markers to other patient groups, we constrained our markers using maps of protein-protein interactions, and we also employed a unique computational strategy that incorporates patient-to-patient molecular variability into the results. We identified a set of 50 genes comprising a *subnetwork signature* that successfully separated GBM patients by their survival times. Our approach to identifying this subnetwork signature also improved our ability to identify its protein products in an independent cohort of patients. In the ongoing search to improve cancer detection and treatment, our work represents a successful strategy for identifying reproducible biomarkers that can more efficiently lead to the discovery of druggable protein targets.

## Introduction

Glioblastoma multiforme is the most common primary brain tumor in adults and, unfortunately, also the most fatal. While GBMs are categorized histologically, the nature of the disease leads to significant variability in both tumor classification and patient outcome. To more specifically define the disease and simultaneously reveal the etiology, an unbiased search for “molecular signatures” of GBM has been undertaken by several groups [1], [2], resulting in a variety of GBM markers which, unfortunately, have modest overlap. Given the large degree of molecular heterogeneity of GBMs, analysis of thousands of patient samples may be required to identify comprehensive gene sets by conventional statistical approaches [3]. However, suggestions that these myriad lists can be integrated via a systems-level analysis, e.g. using molecular networks to find consensus marker sets [4], may help to simplify the observed heterogeneity. In such an approach, an individual gene can affect the algorithmic contribution of a neighboring gene when they coexist in pathways or networks that act to integrate molecular heterogeneity.

While approaches measuring gene expression across a group can capture gene interaction effects, they often employ summary measures, e.g. averaging, that omit valuable information regarding inter- and intra- patient differences. In this work, we hypothesize that the considerable patient-to-patient variability of GBM can be simplified into molecular networks by identifying molecular “state functions” using the computational method, CRANE (for *C*ombinatorially dys*R*egul*A*ted sub*NE*tworks) [5]. The use of molecular states – where the binary expression pattern of a gene set is considered as a whole – allows us to identify subsets of genes whose *configuration* (i.e. the expression pattern rather than expression level alone) distinguishes between the two phenotypes of interest. In this approach, we do not assign a single expression state to a phenotype, but, rather, we search for the set of all states matching a particular phenotype. These expression states are grounded in well-known sets of biological interaction data, as defined by curated protein-protein interaction (PPI) networks.

We applied CRANE to the gene expression data collected by The Cancer Genome Atlas [6] for patients with primary (de novo) GBM. We identified novel subnetwork signatures of survival, which we then tested against an independent gene expression dataset. We also hypothesized that mRNA dysregulation analyzed in the context of PPI subnetworks more efficiently translates to detectible dysregulation at the protein level. To test this, we examined protein expression of selected targets using label-free proteomics in a retrospectively selected set of GBM tumor samples. The workflow presented here is a prototype for identifying manageable subsets of genomic and proteomic targets to ultimately drive the design of cost-effective clinical assays for predicting patient survival – a much desired endpoint for clinicians and patients alike.

## Results

### Subnetwork Signature Discovery

**Figure 1:**
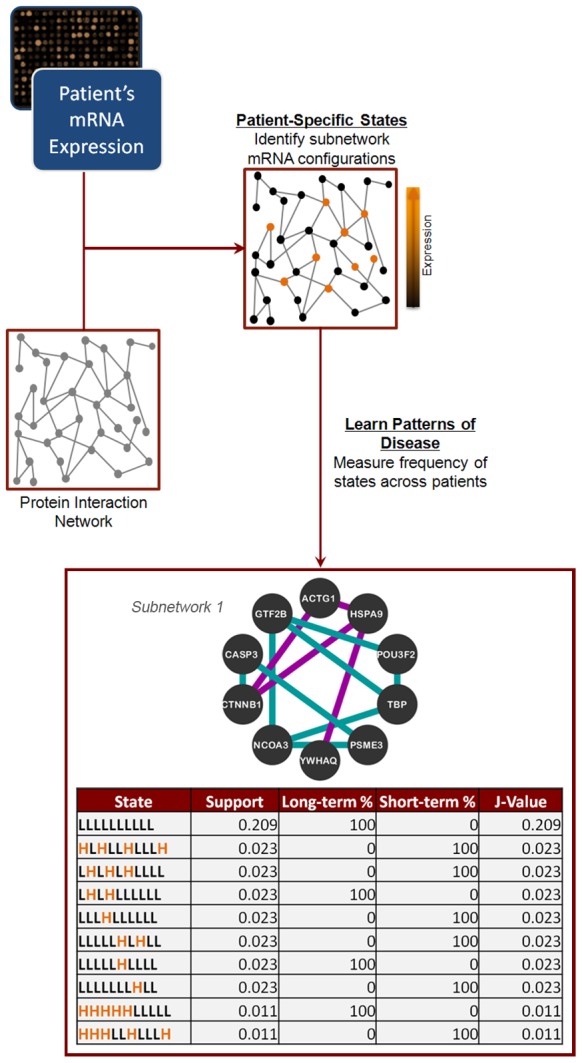
Workflow of the CRANE algorithm for detecting combinatorially dysregulated subnetworks. We begin by mapping patient-specific, binarized mRNA expression data onto a protein interaction network. Then, we identify subnetworks whose pattern of expression – the subnetwork state function – can separate short-term and long-term survivors. Measures of separation are the support (the fraction of samples containing a particular subnetwork state), the fraction of long/short-term survivors, and the *J*-value (see text for description). In the table (bottom), the top ten states of the first TCGA subnetwork are shown. Each row represents a different state of the subnetwork. Each character in the state function (first column) represents the expression state of a particular gene in the subnetwork, where “L” and “H” stand for “low” and “high” expression, respectively.

We began by using GBM patient information and microarray data from The Cancer Genome Atlas [6] (TCGA) as compiled by Verhaak et al. [7]. CRANE, an established method for mining molecular networks [5] (illustrated in Figure 1), successfully identified several subnetworks that were informative in separating short-term (STS) from long-term survivors (LTS) using TCGA mRNA data. The expression patterns for individual genes comprising the top ten states within subnetwork 1 are shown in Figure 1, illustrating how the varied configurations of an individual subnetwork drive the identification of specific subgroups of patients. As an example, note that subnetwork state 3 (LHLHLHLLLL) occurs in two short-term survivors, whereas subnetwork state 4 (LHLHLLLLLL) occurs in two long-term survivors; though state 3 and state 4 differ only in the switch of one gene from *H* to *L*, they predict opposite outcomes. Also, note that the top ten states using these 10 targets only capture 39% of the total patients, reflecting the significant heterogeneity at the patient level. The complete list of subnetwork signature genes can be found in Table S1.

### Subnetwork Signature Testing

**Figure 2:**
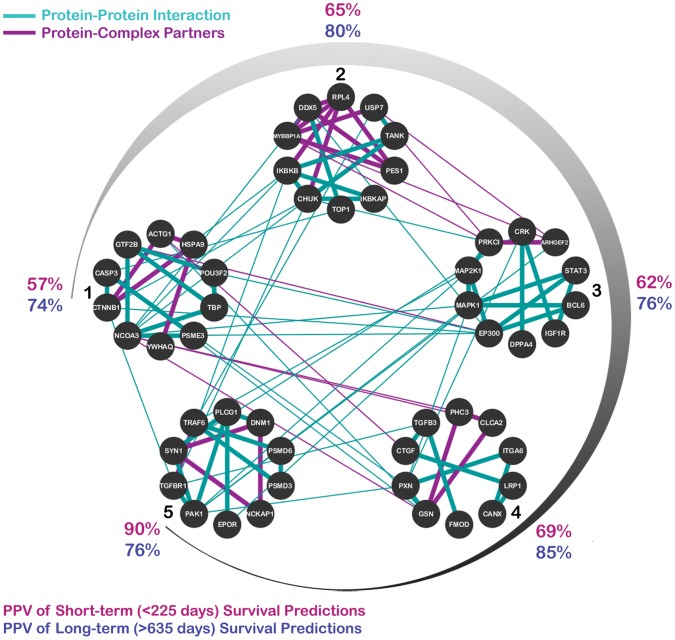
The top five CRANE subnetworks representing a signature of survival in glioblastoma. Gene names are indicated within the nodes; edges represent either protein-protein interactions (turquoise), or proteins found together as partners within a complex (violet). Subnetworks are added into the classifier in clockwise fashion (from 1 to 5); after the addition of each subnetwork, an updated positive predictive value (PPV) is calculated, as shown along the periphery for prediction of both short-term (pink) and long-term (purple) survival.

To investigate the reproducibility of CRANE subnetworks in predicting survival, we tested the TCGA-discovered subnetworks' classification performance on an independent GBM dataset published by Lee et al. [8]. In this analysis, subnetwork discovery and training of the classifier was done on the TCGA data, and testing of the classifier was done on the Lee et al. data. In this test of the TCGA training set, the targets were fixed by the training data (Table S1), and classification accuracy on the Lee et al. data was incrementally calculated for each 10-gene subnetwork (see Methods). We achieved a maximum classification accuracy of 80% when using the top 5 subnetworks generated by CRANE from TCGA data (Figure 2, further details in Table S2); we henceforth refer to this 50 gene set as the *subnetwork signature*. With only 1 subnetwork, or 10 genes, the positive predictive value (PPV) of short-term survival is slightly better than random chance (57%) while the PPV for long-term survival is 74%. The PPV for short-term survival reaches 90% with 5 subnetworks while the maximum observed PPV for long-term survival was 85% with four subnetworks. The cumulative value of using multiple networks – each with a defined set of states – is illustrated in Figure 1. For example, state 1 in sub-network 1 (LLLLLLLLLL) is seen in 21% of patients, while the next 9 states cover only an additional 18% of patients (total of 39% for the top ten states). Thus, the heterogeneity of the patient population cannot be captured even with 10 binarized states from a single subnetwork; multiple subnetworks (each with multiple states) are needed to provide adequate patient coverage and clinically useful prediction accuracy.

### Analysis of Known GBM Molecular Subtypes

Known molecular subclasses of GBM exhibit differences in survival [9], [10], and we examined whether our subnetwork signature was acting as a surrogate for known subtypes. A well-accepted basis for the molecular subtyping of GBMs was recently established by Verhaak et al. using an 840-gene signature [7]. Only four of our top 50 CRANE targets – phospholipase C (PLCG1), paxillin (PXN), transforming growth factor beta 3 (TGFB3), and topoisomerase (TOP1) – overlap with this list, strongly suggesting that our subnetwork signature is not classifying patients by these existing subtypes. Since the CRANE targets may be acting as proxies for the 840 genes, we also checked for an association between our predefined survival groups and molecular subtype using the molecular subtype calls made by Verhaak et al. for the 173 “core” TCGA samples (i.e. those samples most representative of a molecular subtype). When using the 50-gene subnetwork signature for classification, our LTS group consisted of 21% Classical, 35% Mesenchymal, 38% Proneural, and 5% Neural samples while our STS group consisted of 13% Classical, 37% Mesenchymal, 42% Proneural, and 8% Neural samples. Using a chi-square test of independence, we found that these molecular subtypes are not significantly associated (*p*-value>0.05) with membership in our survivor groups in the TCGA data.

**Figure 3:**
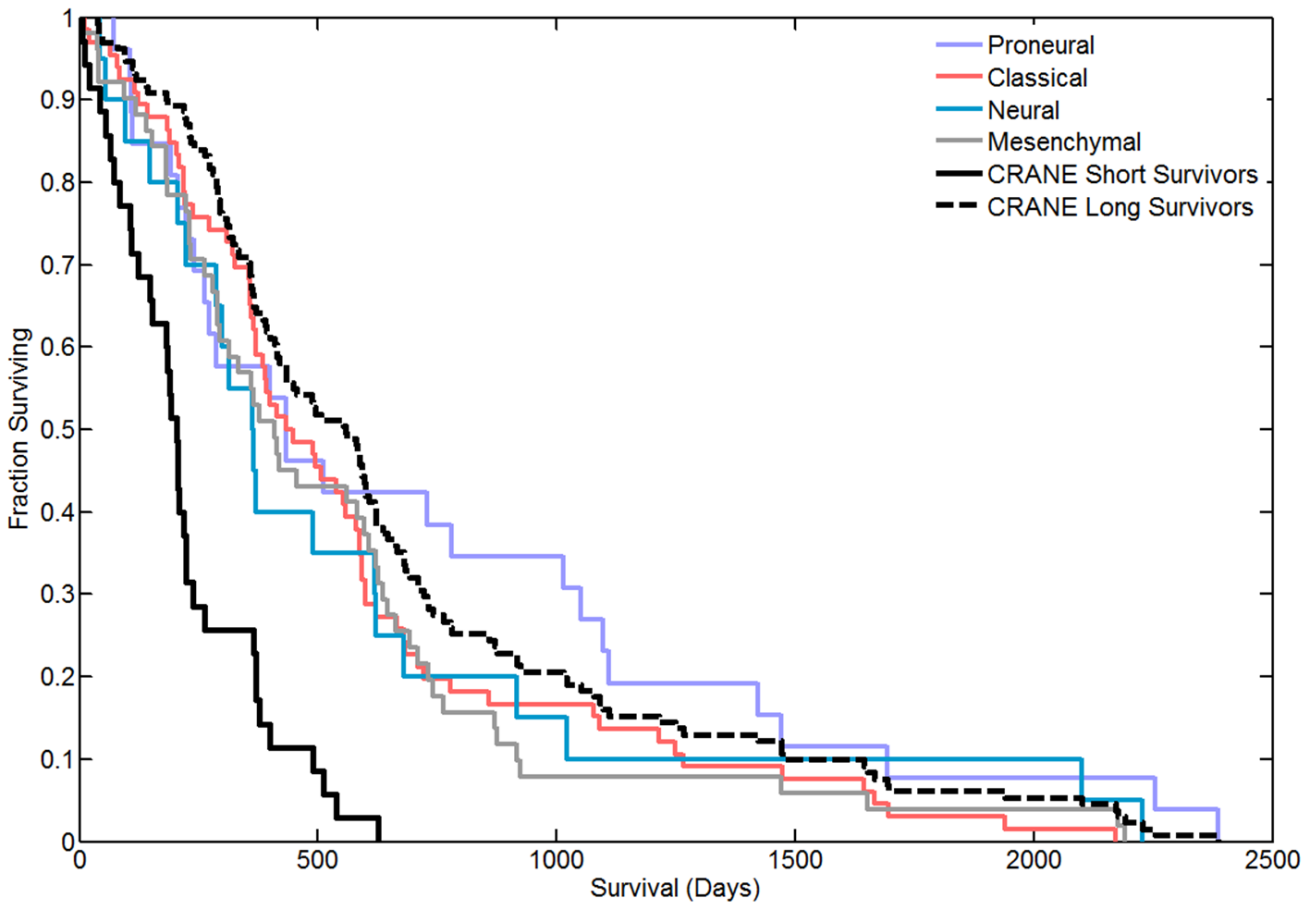
Survival curves comparing various classifiers when tested on the dataset of Lee et al. (GEO ID: GSE13041). While the Verhaak subtypes – Proneural, Classical, Neural, and Mesenchymal – do not show statistically significant differences in survival, the top 5 CRANE subnetworks clearly distinguish short-term from long-term survivor groups.

To examine the extent to which our subnetworks were capturing true differences in survival, we investigated the concordance between the predictions of the network-based classifier and the survival times of the 166 patients in the Lee et al. dataset. As seen in Figure 3, significant differences in survival are apparent between patient groups predicted by the 50-gene subnetwork signature (*p*-value<1e-6, logrank test), indicating the expected performance of the CRANE classifiers within the test dataset. We then compared CRANE's performance against the four subtypes proposed by Verhaak et al. Though the Verhaak signatures were not designed to segregate patients by survival, the Proneural subtype has slightly longer survival than the other subtypes (Figure 3). By the logrank test, there is no significant difference among the four Verhaak subtype survival curves; the four subtypes track the survival curve of the CRANE long-term survivors while the curve for the CRANE short-term survivors is quite distinct.

Given that younger patients tend to have better prognosis [11], we also tested for differences in the age distributions of the two CRANE predicted groups of patients. The age distributions of patients classified by the 50-gene subnetwork signature were similar (Figure S3), and a logrank test indicated that there is insufficient evidence to conclude that the age distributions differ (*p*-value = 0.14). Overall, the above tests show that our CRANE gene expression subtypes are distinct from the Verhaak subtypes and represent novel, age-independent subtype classifications for GBM.

### Subnetwork Signature Proteomics Validation

CRANE examines heterogeneity at the mRNA level to produce state-based classifiers, and we hypothesized that the identified subnetworks transduce this heterogeneity into protein-level differential expression. We tested this hypothesis by examining protein-level changes in an independent cohort of 16 patients from the Ohio Brain Tumor Study, 10 of which were STS and 6 were LTS based on the criteria outlined above. We employed a label-free proteomic approach using ultra-long chromatographic gradients, which permitted the accurate identification and quantification of 5019 peptides from 1491 proteins across the patient samples. Differential expression of proteomic targets was defined using a mixed model of peptides, and we report *p*-values for the differential expression of each protein. Using this model, 338 proteins were significantly up- or down-regulated at a *p*-value≤0.05 (Table S3). We did not make false-discovery rate corrections for these *p*-values as this is not an unbiased discovery experiment. Instead, we were interested in modeling how proteomic expression varied for pre-specified subsets of genes. Although proteomics has less dynamic range than gene expression analysis, the above method permitted the confident identification and quantification of over one-third of the CRANE subnetwork signature (17/50 targets). Of the 17 targets of interest that were identified and measured (see Table 1), five proteins were significantly down-regulated and two were significantly up-regulated in LTS. Interestingly, these 7 proteomic targets have modest classification potential at the level of individual gene expression, as illustrated by the irregularity of their gene expression patterns in the TCGA dataset (Figure S2).

**Table 1:** Dysregulated proteins identified within the 50-gene subnetwork signature.

IPI ID	Protein Description	# of peptides	STS mean	LTS mean	Ratio (LTS/STS)	*p*-value
IPI00784414.1	STAT3 Isoform Del-701 of Signal transducer and activator of transcription 3	1	0.25	−0.42	0.64	0.200
IPI00010697.2	ITGA6 Isoform Alpha-6X1X2B of Integrin alpha-6	1	0.16	−0.27	0.65	0.416
**IPI00003918.6**	**RPL4 60S ribosomal protein L4**	**2**	**0.29**	**−0.48**	**0.72**	**0.029**
**IPI00026314.1**	**GSN Isoform 1 of Gelsolin**	**14**	**0.17**	**−0.29**	**0.73**	**5.29E-04**
**IPI00020984.2**	**CANX Calnexin**	**9**	**0.25**	**−0.42**	**0.74**	**3.77E-05**
IPI00607584.1	MYBBP1A Isoform 2 of Myb-binding protein 1A	2	0.15	−0.24	0.77	0.289
IPI00017617.1	DDX5 cDNA FLJ59357, highly similar to Probable ATP-dependent RNA helicase DDX5	3	0.15	−0.25	0.79	0.167
**IPI00011603.2**	**PSMD3 26S proteasome non-ATPase regulatory subunit 3**	**4**	**0.18**	**−0.31**	**0.80**	**0.052**
**IPI00007765.5**	**HSPA9 Stress-70 protein, mitochondrial**	**13**	**0.22**	**−0.37**	**0.80**	**1.34E-05**
IPI00018146.1	YWHAQ 14-3-3 protein theta	5	0.16	−0.27	0.86	0.056
IPI00020557.1	LRP1 Prolow-density lipoprotein receptor-related protein 1	9	0.08	−0.14	0.91	0.185
IPI00472160.5	ARHGEF2 Isoform 1 of Rho guanine nucleotide exchange factor 2	1	−0.01	0.02	1.01	0.957
IPI00017292.1	CTNNB1 Isoform 1 of Catenin beta-1	4	−0.07	0.12	1.09	0.449
IPI00409684.2	NCKAP1 Isoform 2 of Nck-associated protein 1	2	−0.19	0.32	1.29	0.156
**IPI00003479.3**	**MAPK1 Mitogen-activated protein kinase 1**	**6**	**−0.22**	**0.37**	**1.50**	**3.78E-03**
IPI00656138.1	PAK1 Isoform 1 of Serine/threonine-protein kinase PAK 1	1	−0.27	0.44	2.14	0.178
**IPI00887273.1**	**DNM1 Isoform 2 of Dynamin-1**	**4**	**−0.25**	**0.42**	**2.18**	**6.45E-03**

Proteins with p-values<0.05 are in bold. Ratios (LTS-to-STS) were calculated from the raw data.

To explore the prognostic potential of the proteomic targets, we used classification and regression trees (CART) to identify patterns of proteins that would robustly classify STS from LTS using the significant proteomic targets; for classification, we used the 7 significantly differentially expressed proteomic targets, as well as YWHAQ, which was of borderline significance. This yielded a simple 2-gene protein-level classifier, illustrated in Figure S4. Using only CANX and MAPK1, the classifier is able to correctly identify 100% of long-term survivors in the group and 90% of short-term survivors. For example, when CANX has a normalized value greater than −1.05 and MAPK1 is less than 0.50, we can identify 9 of our short-term survivors, though such high sensitivity and specificity are likely indicative of over-fitting.

To explore our hypothesis that the use of network topology improves our ability to detect targets at the protein level, we compared the performance of CRANE-identified targets versus that of individual gene markers in identifying dysregulated proteins. The “individual gene markers” refers to a set of the most differentially expressed genes selected without respect to any underlying interaction structure. Specifically, we identified all genes with a fold-change ≥2 between the 86 LTS and STS survivors in the TCGA data and then ranked these genes according to their absolute t-statistic (i.e. the difference in group means divided by the pooled standard deviation). Of the top 200 individual gene markers, only one – ACTG1 – overlapped with the 50-gene subnetwork signature. Thus, 49/50 genes identified using a network-based classifier could not be discovered based on conventional analysis of individual gene markers.

**Figure 4:**
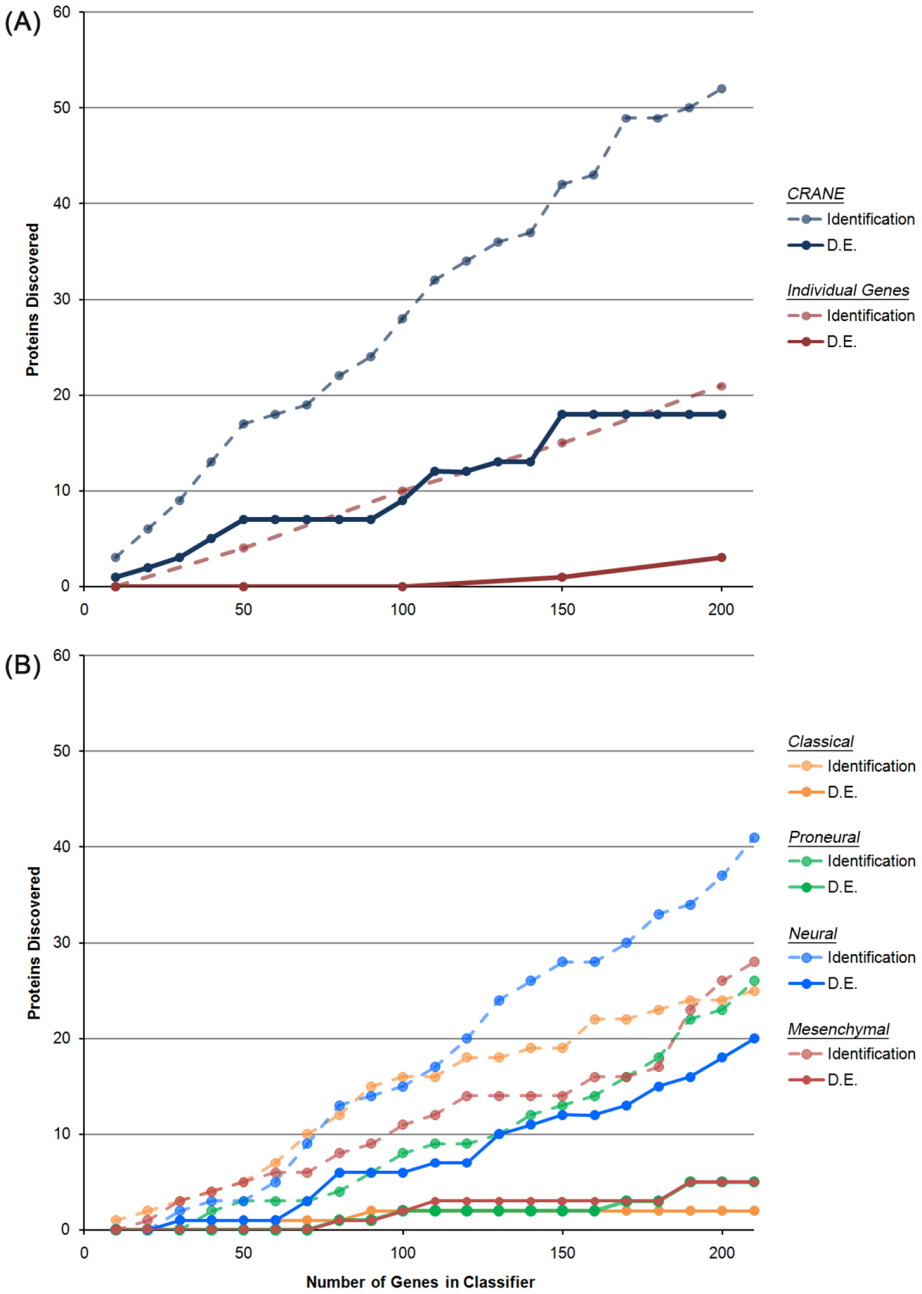
Proteomic detection and dysregulation of biomarkers discovered using various pipelines. (A) Comparison of the number of proteomic targets identified using a network-based algorithm for identifying combinatorial gene markers (“CRANE”) versus one using individual differentially expressed genes (“Individual Gene Markers”). (B) Comparison of the number of proteomic targets identified using the subtypes identified by Verhaak et al. We plot the total number of classifier targets detected in the proteomic experiment (“Identification”), as well as the subset of classifier genes showing evidence for differential expression (*p*-value≤0.05) at the protein level (“D.E.”).

As seen in Figure 4A, the use of an interaction network in an mRNA-based classifier markedly improves our ability to identify targets differentially expressed at the protein level compared to examination of individually dysregulated genes. Specifically, CRANE identified dysregulated subnetworks that were better represented in the proteomic data, and these subnetworks included more differentially expressed proteins when compared to dysregulated individual gene markers. When interrogating the proteomics data for the top 200 network-based genes (i.e. the top 20 CRANE subnetworks), over 50 proteins were identified (25%) and 18 of these subnetwork proteins showed differential expression (36% differentially expressed among those identified). In contrast, when using the top 200 differentially expressed individual genes, 21 were identified via proteomics (10%) and only 3 showed significant changes (14% differentially expressed among those identified). Fitting a linear regression model to the data, we find that individual gene markers yield differentially expressed proteins at a rate of 1.5% (relative to the number of genes used), whereas the network-based approach has a rate of return of 9.8% - a 6.5-fold improvement in the yield of our proteomics validation experiment.

We also explored the proteomic yield of the four Verhaak et al. subtypes. As shown in Figure 4B, the 210-gene Neural subtype had the best yield in the proteomics experiment, with 41 targets identified via proteomics (20% of all targets identified) and 20 showing significant changes (49% differentially expressed among those identified). However, the number of proteomic targets identified by the Proneural, Classical, and Mesenchymal subtypes was considerably lower. While the rate of return for these three subtypes (ranging from 1%–2.7%) was comparable to that of the individual gene markers, the rate of return for the Neural subtype was 9.9%.

## Discussion

In this work, we analyzed the mRNA-level heterogeneity of GBMs using protein interaction networks, arriving at a succinct list of 50 genes that predicts patient survival at 80% accuracy. Not only does the unique subnetwork signature show reproducible prediction of patient survival at the mRNA level, it also exhibits protein-level dysregulation that segregates short-term from long-term survivors of glioblastoma – a valuable characteristic in light of recent evidence suggesting that many mRNA-level signatures have questionable classification power and modest biological significance [12]. Additionally, the 50-gene subnetwork signature indentified here represents an experimentally tractable number of targets – measurable in a streamlined proteomics experiment – while previously discovered target lists are not likely to be translated into clinical assays due to their large size [7]. While past work on unsupervised classification of high-grade gliomas was complicated by the use of mixed WHO grade III and grade IV patient samples [1], [13]–[16], we herein develop a molecular signature based solely on primary, untreated grade IV tumors from the TCGA database. We note the caveat that the number of subnetworks included in the signature was selected based on the classification performance on the test (Lee et al.) data and, thus, requires further validation to be useful as a standalone classifier of gene expression data. In this work, we choose, instead, to explore how this 50-gene subnetwork signature behaves at the protein level.

Building upon the success of gene pair classifiers [17], the network analysis framework presented here identifies multigene subnetworks based on mRNA state functions – series of 1's and 0's – allowing us to account for patient-level heterogeneity in expression profiles. While binarization of continuous expression data certainly involves a loss of information, this concept lends itself to the design of therapeutic interventions, where targeted molecular therapies inhibit or activate key “switches” in the circuits of distinct patient subtypes. For instance, upregulation of insulin-like growth factor receptor (IGF1R), seen in subnetwork 3, has been identified in a wide variety of human cancers [18], and *in vitro* evidence suggests that this upregulation contributes to resistance against EGFR inhibitors [19]. Our results suggest that IGF1R has variable expression – on, or 1, in some tumors and off, or 0, in others – in patients within the same GBM survival class, indicating that experimental IGF1R monotherapies [20], [21], while inappropriate as a population-level intervention, may be highly effective in precisely selected individuals. A binary model of expression-activation is an oversimplification in some instances, however, where protein activity does not necessarily correlate with expression levels, e.g. in the case of kinases.

In contrast to proteomic approaches, several groups have worked on classifying the genomic alterations underlying GBM [22], [23]. Of the 309 unique, validated mutations identified through sequencing of the TCGA GBM tumor samples, *CTNNB1*, *EP300*, *STAT3*, and *TOP1* also appear in the 50-gene subnetwork signature. These genomic alterations are likely to play causative roles in establishing the global state function of the subnetwork signature. β-catenin (*CTNNB1*), for instance, complexes with N-cadherin to coordinate tumor invasiveness [24] and shows some promise as a prognostic marker [25]. Additionally, *TOP1* is targeted by topoisomerase inhibitors to treat a wide variety of cancers [26], [27]. CRANE identifies these key genes not simply because they show consistent expression across a group, but, rather, because their expression levels form a distinct pattern when viewed in conjunction with the 46 other genes in the milieu. This is in line with the known patient-to-patient variability in the mutational landscape of cancer [28]. In this light, the presence or absence of common mutations in patient subgroups differentially disrupts network state functions, and a single chemotherapeutic agent is unlikely to be effective in every patient.

We hypothesized that the underlying network structure would ultimately lead to differences in protein expression between survival groups. Using a mixed model accounting for inter-peptide dependencies within a protein, we identified 7 dysregulated proteins out of a total of 17 detected in the proteomics experiment from the 50-gene subnetwork signature. Though the stochastic nature of proteomics workflows may have discouraged their use as validation platforms, we demonstrate that ultra-long chromatographic gradients coupled with high-resolution mass spectrometers allow us to probe the signaling networks of interest in a high-throughput fashion, with chromatographic reproducibility (Figure S1) sufficient for the development of targeted assays (i.e. using pre-specified lists of M/Z values to measure daughter peptides of network targets).

To gauge how the interaction network influenced our success in identifying dysregulated protein targets, we compared the proteomic performance of CRANE against that of a signature based on differentially expressed individual genes. We found a marked improvement in our ability to detect protein-level changes in identified markers when a network-guided combinatorial algorithm is used to detect mRNA-level dysregulation signatures (see Figure 4), and the improved representation of subnetwork targets in the proteomic data can be attributed, in part, to the use of the PPI network. Sources of experimental bias in the measurement of protein expression can be similar to those in the identification of PPIs (i.e. more abundant proteins are more easily identified). However, when we consider the fraction of differentially expressed proteins among all proteins identified, the top 200 CRANE targets always deliver more than 30% precision in identifying differentially expressed proteins, reaching a maximum of 43% when 150 targets are evaluated. In contrast, when we consider the products of the top 200 individual gene markers (i.e. those having significant mRNA differential expression), the fraction of differentially expressed proteins reaches a maximum of only 14%. Assuming the trend in discovery is linear, the network-based approach affords a nearly 7-fold improvement in the rate of discovery of differentially expressed proteins. As a testament to the combinatorial aspect of our analysis, our seven differentially expressed proteomic targets (in Table 1) would not have been discovered if we had based our classifier on individually differentially expressed genes, for these proteins did not exhibit consistent mRNA expression across survival groups in the TCGA data (Figure S2). While it is well known that dysregulation at the level of individual gene expression does not necessarily correlate with protein expression (the mRNA-to-protein correlation is 0.43 for humans [29]), our observations clearly suggest that combinatorial, network-based mRNA-signatures serve as better indicators of post-transcriptional dysregulation when compared to sets of differentially expressed single genes. This result speaks to the ability of network-based algorithms to reproducibly detect dysregulated proteins at the population level, as opposed to uncovering the relationship between mRNA expression and protein expression within a single sample. As an alternative explanation, the network-based targets may point to proteins that are more abundantly expressed and for which dysregulation can be more efficiently measured.

Given that the Verhaak et al. subtypes were constructed through hierarchical clustering of gene expression data, we expected that their yield in a proteomics experiment would largely compare to the performance of individual gene markers (which were constructed based on ranked differential expression). While this was the case for Proneural, Classical, and Mesenchymal subtypes, the Neural subtype performed relatively well in predicting differentially expressed proteins, yielding proteomic targets at a rate comparable to the CRANE signatures. This suggests that the Neural subtype contains hidden network structure that boosts the visibility of the group at the protein level and/or that both the CRANE signature and the Neural subtype contain classes of proteins (e.g. structural and metabolic proteins) that are more amenable to proteomic measurement. In support of the latter hypothesis, the top gene ontology (GO) term in the Neural subtype was nucleotide metabolic process (GO:0009117, *p*-value = 4.72e-5) [7], and metabolic enzymes are typically well-represented in proteomic experiments [30].

We also examined gene ontology (GO) term enrichment of our CRANE signature using DAVID [31], and we compared the results to the enrichment of the Verhaak et al. subtypes. Of the CRANE GO terms significant at the 0.01 level, only 6 overlapped and were significant (*p*-value≤0.01) in the Verhaak et al. dataset, including terms such as “regulation of transcription,” “regulation of cell proliferation,” and “cytoskeletal organization” (see Table S4 for the complete list of significant overlapping terms). The most significant and informative GO terms found in the CRANE signature included items such as “protein kinase cascade” (GO:0007243, *p*-value = 3.98e-8), “I-kappaB kinase/NF-kappaB cascade” (GO:0007249, *p*-value = 6.56e-5), and “regulation of programmed cell death” (GO:0043067, *p*-value = 8.08e-5), all of which were absent or not significant in the Verhaak et al. subtypes (see Table S5 for the complete list of terms significant in the CRANE signature). These results indicate that the CRANE subnetwork signature emphasizes kinase cascades and the NF-κB pathway. NF-κB expression has been shown to be positively correlated with astrocytoma grade and inversely correlated with patient survival [32]. Importantly, deletions of NF-κB inhibitor α (NFKBIA) and amplifications of EGFR have been shown to be mutually exclusive events in GBM [33], suggestive of underlying genomic subtypes. Our work recapitulates the importance of understanding patient-to-patient variability in NFKB signaling to better direct therapeutic decisions.

Seven subnetwork targets were validated using proteomics, and these proteins have interesting connections to both glioma and cancer. For example, HSPA9 is not only upregulated in a variety of cancers [34], [35], but its expression also correlates with glioma grade and the proliferative potential of cells [36]. In our data, HSPA9 is strongly (fold change = 0.80) and significantly (*p-*value = 1.34e-5) downregulated in the tumors of long-term survivors, suggesting that, even between tumors of the same grade, HSPA9 biology may differentially affect patient survival. Similarly, we found that calnexin (CANX) has 0.74-fold diminished protein expression in long-term survivors, and this result is in line with the observation that CANX expression is significantly correlated with the transition from angiogenesis-independent to angiogenesis-dependent (i.e. more invasive) tumor growth in xenografts [30]. In turn, PSMD3, a subunit of the 26S proteasome, was also found to be downregulated in the tumors of long-term survivors, which is in line with the promising results of proteasome inhibitors in pre-clinical studies [37], [38]. More recently, a novel role for PSMD3 was proposed by Okada et al., who identified a SNP near the gene associated with the regulation of neutrophil count by both GWAS and eQTL analysis [39]. It has long been recognized that cancer and inflammation are synergistic processes [40], and it appears that increased neutrophil activity is associated with highly infiltrative gliomas [41], [42]. Given the potential role of PSMD3 in neutrophil recruitment in GBMs, our data are consistent with a hypothesis that downregulation of PSMD3 leads to less neutrophil-mediated inflammation and longer survival.

In assessing patient outcomes of GBM, we argue that the most informative prediction is whether or not a patient has a poor prognosis, i.e. is a “short-term survivor,” as this prognosis identifies patients who are poor candidates for the standard of care and for whom more aggressive therapies may be beneficial. To demonstrate the therapeutic potential of proteomic targets, we used CART to identify a decision tree useful in classifying our proteomic cohort. We found that two proteins could effectively classify our cohort of 16 patients with near perfect sensitivity and specificity, though this result may be due to overfitting in our cohort. Nonetheless, this result illustrates how gene expression targets may be translated into clinical proteomics biomarkers.

We note that the many of the GBM patients with a poor prognosis in our proteomic validation cohort did not receive the full standard of care: surgery, radiation, and chemotherapy. Consequently, survival classification in our study is not a proxy for response to the standard of care. In future clinical work, efforts should be directed to identifying cancer survivors matched on treatment protocols to allow for the identification of molecular features that render them susceptible to various therapies. While our 50-gene network signature is currently useful for prognostication, analysis of a treatment-matched cohort would potentially allow for the identification of targets to guide therapeutic decision making.

## Methods

### Source Data

The results published here are in part based upon data generated by The Cancer Genome Atlas (TCGA) pilot project established by the NCI and NHGRI. Information about TCGA and the investigators and institutions who constitute the TCGA research network can be found at http://cancergenome.nih.gov/. Patient data was obtained from TCGA, where clinical data and corresponding microarray data were available for 200 glioblastoma patients [6]. Samples run on three different array platforms – the Affymetrix U133A GeneChip, the Affymetrix Human Exon GeneChip, and a custom-made Agilent array – were pooled into a composite dataset by Verhaak et al. [7], and these data were used for further analysis. To select only *de novo* GBM, we removed those patients with a pretreatment history, a histologic classification of “treated primary GBM”, or a prior history of glioma. We also excluded patients whose final vital status (living vs dead) was unknown. The remaining patients were separated into two groups based on survival, taking the top 25% (43 patients, surviving>635 days, ages 11–83) as long-term survivors and the bottom 25% (43 patients, surviving<225 days, ages 39–85) as short-term survivors.

### Subnetwork Signature Discovery

CRANE [5] was employed to discover subnetworks of proteins coordinately dysregulated at the level of mRNA; the MATLAB code is available. The global human protein-protein interaction network was compiled from publicly available interactions in the Human Protein Reference Database [43], and the CRANE search algorithm was constrained to subnetworks of consisting of at most 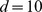 proteins. We binarized gene expression data by setting the genes in the top quartile of expression intensity to H (high expression) and all others (bottom 75%) to L (low expression). This threshold for high expression (25%) was previously shown to be most effective in identifying discriminative subnetworks using a range of datasets [5]. After binarizing the data, we were interested in identifying subnetworks whose “state” – the binary sequence of H's and L's – was informative in regards to the phenotype (STS vs LTS). This is formulated as an optimization problem, where the objective function to be maximized is the mutual information between phenotype and expression state, the *J-*value. Mutual information is a measure of the reduction in our uncertainty of a patient's phenotype, given observations of the subnetwork's expression state. More precisely, denoting the phenotype random variable with 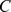 and letting 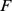 denote the *k*-dimensional binary random variable representing the expression state of a subnetwork of size *k*, the mutual information between the expression state and phenotype is defined as 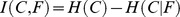. Here, 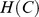 denotes the entropy of the phenotype random variable, and 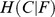 denotes the entropy of the phenotype given the expression state of the subnetwork, 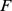. The entropy of a random variable X is defined as 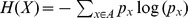, where *A* denotes the set of all possible values of *X* and *p_x_* denotes 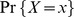.

We refer to a particular expression state of a particular subnetwork as a “state function.” For a state function, the *J-*value is defined as the amount of information provided by that particular state on the phenotype, i.e. its contribution to the mutual information between phenotype and the state of the corresponding subnetwork. Namely, for a given state function *f* for a subnetwork composed of *k* proteins (i.e., *f* is an observation of random variable 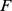), the *J*-value is defined as 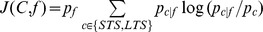. Here, 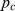 denotes 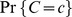, and 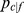 denotes 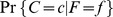. It can be shown that 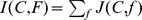.

In this analysis, we first identified high-scoring subnetworks according to their *J*-values and then sorted these high-scoring subnetworks according to their mutual information for survival. Additional parameters used to assess a network's prediction accuracy are the support (the fraction of samples containing a particular subnetwork state, 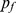); the confidence (the fraction of long-term survivors possessing a particular subnetwork state, 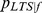); and the anti-confidence (the fraction of short-term survivors possessing a particular subnetwork state, 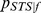). A subnetwork and an associated state function have a high *J*-value if the state function provides high support, high confidence, and low anti-confidence (or, symmetrically, high anti-confidence and low confidence).

### Subnetwork Signature Testing

To test the network features discovered using TCGA, we explored their prediction accuracy using an independent GBM microarray dataset, GSE13041, available via the Gene Expression Omnibus [8]. After removing patients known to have received prior radiotherapy, chemotherapy, and/or temozolomide treatment, a total of 166 patients remained; using the survival time cut-offs as before, the short-term survivor group consisted of 41 patients (ages 34–86), and the long-term survivor group consisted of 50 patients (ages 22–78).

A neural network (NN) was trained on the TCGA data using the top *k* subnetworks (ranked by mutual information, where *k* is a variable), and test performance was gauged using classification accuracy, calculated as 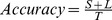, where *S* is the number of correctly predicted short-term survivors, *L* is the number of correctly predicted long-term survivors, and *T* is the total number of test samples in the test dataset. We calculated the cumulative classification accuracy for *k* ranging from 1 to 10, i.e. examining accuracy of the best performing network alone, and then examining the performance of the best two networks, and then the best three networks, etc. Overall classification accuracy reached a maximum of 80% when using *k* = 5 subnetworks, each composed of size *d* = 10 genes (Table S2).

For comparison, we assessed how the four GBM subtypes proposed by Verhaak et al. stratified patient survival in the testing dataset, GSE13041. We first removed pretreated patients from the testing dataset, and the data was then log transformed, median centered, and normalized by each array's standard deviation; gene expression was inferred by averaging probe-level expression. For the 840 genes in the Verhaak et al. GBM subtype classifier, we calculated the Spearman correlation coefficient between the centroid expression profiles (derived from the TCGA dataset) and each sample in the testing dataset, assigning each sample to the subtype with maximum correlation.

### Patient Description for Proteomics

#### Ethics statement

The Case Comprehensive Cancer Center Institutional Review Board approved this study at a full board review.

For proteomic analysis, tumor samples were obtained retrospectively from 18 patients diagnosed with GBM surgically resected at University Hospitals Case Medical Center, with no prior radiotherapy or chemotherapy. Brain tumor tissue was snap frozen 15–30 minutes post tumor resection. One patient was removed as they had an incorrectly documented length of survival; a second patient was removed as their sample's liquid chromatography-mass spectrometry (LC-MS) profile was classified as an outlier by principle component analysis. Of the remaining 16 patients, ten patients had survival time of 9 months or shorter (short term survivors; STS) and 6 patients had survival time of 18 months or longer (long term survivors; LTS). Average survival time in months for STS was 4.8 months and for LTS was 25.5 months. Both survival groups had the same proportion of females (60%) and similar average age at diagnosis (64.3 years for STS; 61.5 years for LTS). The STS had an average post-operative Karnofsky Performance Score (KPS) of 63.3 while the LTS had an average KPS of 80. All LTS patients received post-operative temozolomide and radiation while only 2 and 7 of the STS received post-operative temozolomide and radiation, respectively.

30–50 mg of tumor sample was rinsed twice with ice cold phosphate buffered saline (Pierce), and lysed in 450 µL 4% sodium dodecyl sulfate buffered with 50 mM Tris; protease and phosphatase inhibitors (Pierce) were also added. After probe sonication and centrifugation, 150 µL of the lysate was reduced with dithiothreitol (10 mM in solution) and alkylated with iodoacetamide (550 mM in solution). The protein fraction was collected through crashing three times with ice cold acetone. After drying, the protein pellet was resuspended in 200 µL 70% trifluoroacetic acid, and protein concentration was quantified using a Bradford assay. We raised the volume of 150 µg of protein to 100 µL and digested the protein overnight using cyanogen bromide (300 µg). After drying the sample, the peptides were resolubilized in 100 µL of 50 mM ammonium bicarbonate and 4 M urea. The sample was vortexed and then diluted to attain a final urea concentration of 1 M. The samples were then digested overnight with trypsin at 37°C. The digestion was quenched with trifluoroacetic acid, and salts were removed using a C18 column (Nest Group). After drying the cleaned eluent, the sample was resolubilized in 100 µL of 0.1% formic acid in preparation for LC-MS/MS. As successful application of a label-free LC-MS/MS approach hinges on robust and reproducible retention times and MS1 intensity stability, an external spike-in of trypsin digested yeast enolase (400 fmole, on-column) was included in all the GBM samples to monitor instrument performance parameters for each LC-MS run; see Figure S1 for representative chromatograms.

After lysing the samples and isolating the protein fraction, the 16 samples were analyzed by LC-MS/MS with 4 hour gradients; raw data processing and normalization was performed in Rosetta Elucidator (see Supplemental Methods for mass spectrometry parameters).

### Statistical Methods

To identify statistically significant proteomic changes, missing values were imputed using the median intensity per peptide within each survival group, and the data was standard normalized for each peptide. We used a mixed model to compare the group-wise protein intensity differences of interest, with the survival group set as a fixed effect and the peptide set as a random effect, which allowed us to account for the within-protein correlation of the peptides inherent in mass spectrometry-based proteomic experiments [44]. In the results, we only compare differences between various prespecified protein sets observed in the data, namely the proteins coded by the following genes: the genes in the top-ranking subnetworks identified by CRANE (200 genes in total), the genes in the Verhaak molecular subtypes (840 genes in total), and the top 200 genes with the most significant individual differential expression. Using a likelihood ratio test, a *p*-value≤0.05 for the proteins of interest was considered significant and no correction for multiple hypothesis testing was performed. These statistical analyses were performed using R 2.13.2 and SAS version 9.2 (SAS Institute Inc., Cary, NC).

## Supporting Information

Figure S1 Retention time plots for three representative proteomic samples illustrating the quality of chromatographic reproducibility. We have shown the 20–220 minute time period out of the total 4 hour run time.
(TIF)

Figure S2 Heatmap of mRNA expression from the TCGA dataset for the 7 differentially expressed proteomic targets. The TCGA tumor samples are rank-ordered by survival, which is shown along the bottom as the number of days till death.
(TIF)

Figure S3 Distributions of the long-term survivors (LTS) and short-term survivors (STS) as defined by the CRANE subnetwork signature. (Top) Survival curves of LTS vs STS. (Bottom) Age distributions of LTS and STS groups. By the log-rank test, there is insufficient evidence to conclude that the age distributions differ (*p*-value = 0.14).
(TIF)

Figure S4 Classification and regression tree for the proteomic targets. Using CANX and MAPK1 alone, all 6 of our long-term (LT) survivors and 9 of our short-term (ST) survivors are classified correctly.
(TIF)

Table S1 List of 50 genes in the subnetwork signature of survival for primary GBM.
(PDF)

Table S2 Classification results of CRANE subnetworks discovered from the TCGA patient cohort and tested on an independent patient dataset (GSE13041). Results reflect classification accuracy of testing incremental combinations of subnetworks (e.g. classification results of Subnetwork 3 represent the use of subnetworks 1, 2, *and* 3 in classifying the independent patient dataset).
(PDF)

Table S3 Differential expression of proteomic targets (*p*-value≤0.05) identified in a cohort of 16 GBM patients.
(XLS)

Table S4 List of significant GO terms overlapping between the Verhaak subtypes and CRANE 50-gene subnetwork signature.
(XLS)

Table S5 List of GO terms significant (*p*-value≤0.05) in the CRANE 50-gene subnetwork signature.
(XLS)
