# Supplementary figures and images for: Network Signatures of Survival in Glioblastoma Multiforme
Source: PLoS Comput Biol. 2013 Sep 19;9(9):e1003237. doi: 10.1371/journal.pcbi.1003237 (PMC3777929; doi:10.1371/journal.pcbi.1003237)

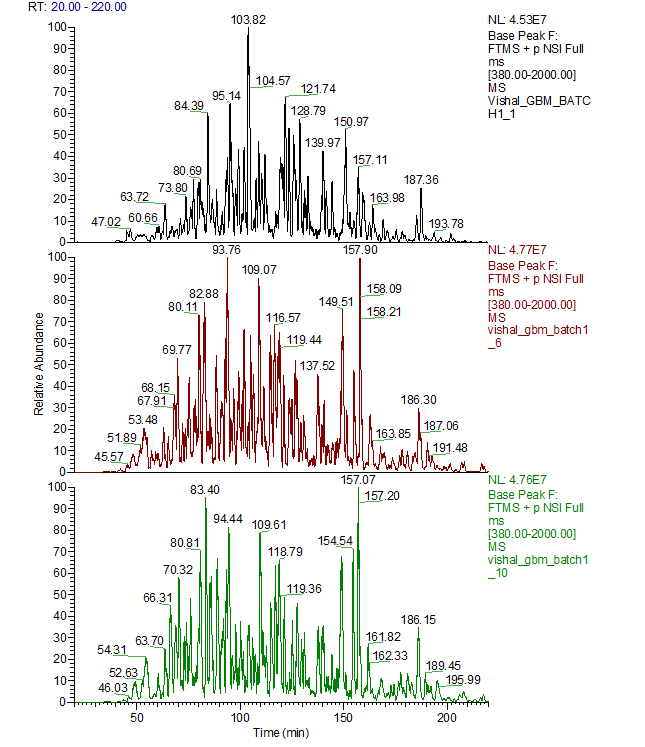

Supplement: Figure S1 — Retention time plots for three representative proteomic samples illustrating the quality of chromatographic reproducibility. We have shown the 20–220 minute time period out of the total 4 hour run time. (TIF) [file pcbi.1003237.s001.tif]

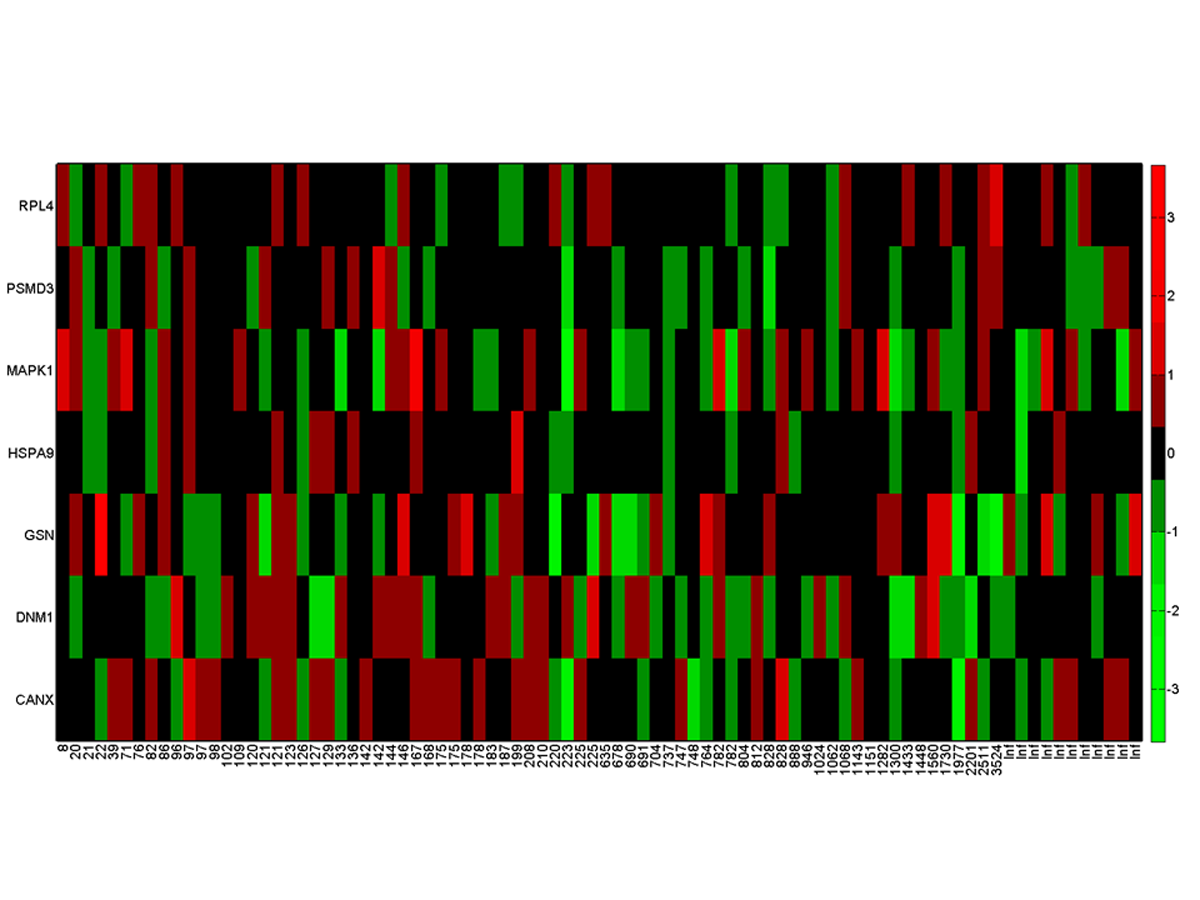

Supplement: Figure S2 — Heatmap of mRNA expression from the TCGA dataset for the 7 differentially expressed proteomic targets. The TCGA tumor samples are rank-ordered by survival, which is shown along the bottom as the number of days till death. (TIF) [file pcbi.1003237.s002.tif]

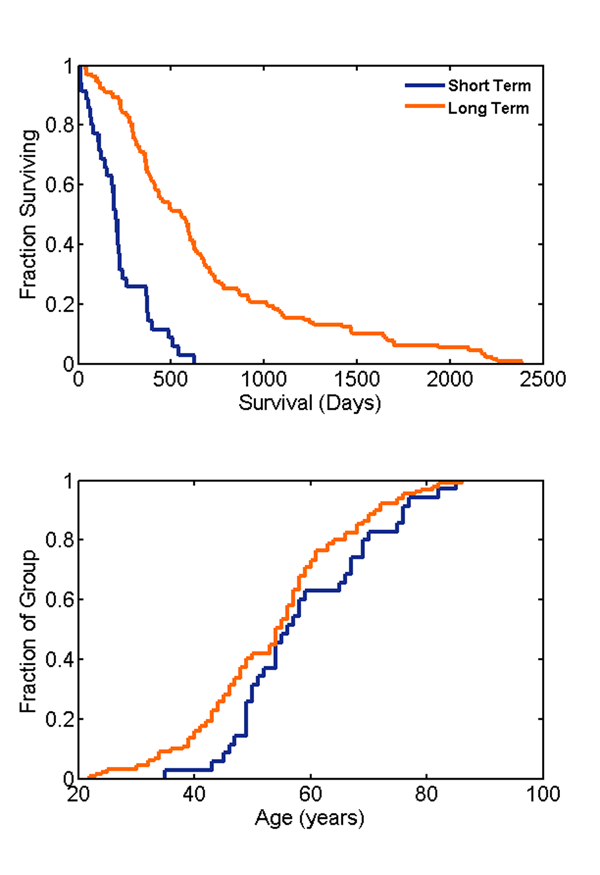

Supplement: Figure S3 — Distributions of the long-term survivors (LTS) and short-term survivors (STS) as defined by the CRANE subnetwork signature. (Top) Survival curves of LTS vs STS. (Bottom) Age distributions of LTS and STS groups. By the log-rank test, there is insufficient evidence to conclude that the age distributions differ (p-value = 0.14). (TIF) [file pcbi.1003237.s003.tif]
