# Supplementary material for: Network Signatures of Survival in Glioblastoma Multiforme
Source: PLoS Comput Biol. 2013 Sep 19;9(9):e1003237. doi: 10.1371/journal.pcbi.1003237 (PMC3777929; doi:10.1371/journal.pcbi.1003237)
Supplement: Table S1 — List of 50 genes in the subnetwork signature of survival for primary GBM. (PDF) [file pcbi.1003237.s005.pdf]

Table S1. List of 50 genes in the subnetwork signature of survival for primary GBM.

Gene Name	Entrez GeneID
**ACTG1**	71
**ARHGEF2**	9181
**BCL6**	604
**CANX**	821
**CASP3**	836
**CHUK**	1147
**CLCA2**	9635
**CRK**	1398
**CTGF**	1490
**CTNNB1**	1499
**DDX5**	1655
**DNM1**	1759
**DPPA4**	55211
**EP300**	2033
**EPOR**	2057
**FMOD**	2331
**GSN**	2934
**GTF2B**	2959
**HSPA9**	3313
**IGF1R**	3480
**IKBKAP**	8518
**IKBKB**	3551
**ITGA6**	3655
**LRP1**	4035
**MAP2K1**	5604
**MAPK1**	5594
**MYBBP1A**	10514
**NCKAP1**	10787
**NCOA3**	8202
**PAK1**	5058
**PES1**	23481
**PHC3**	80012
**PLCG1**	5335
**POU3F2**	5454
**PRKCI**	5584
**PSMD3**	5709
**PSMD6**	9861
**PSME3**	10197
**PXN**	5829
**RPL4**	6124
**STAT3**	6774
**SYN1**	6853
**TANK**	10010
**TBP**	6908
**TGFB3**	7043
**TGFBR1**	7046
**TOP1**	7150
**TRAF6**	7189
**USP7**	7874
**YWHAQ**	10971
