# Supplementary material for: Network Signatures of Survival in Glioblastoma Multiforme
Source: PLoS Comput Biol. 2013 Sep 19;9(9):e1003237. doi: 10.1371/journal.pcbi.1003237 (PMC3777929; doi:10.1371/journal.pcbi.1003237)
Supplement: Table S2 — Classification results of CRANE subnetworks discovered from the TCGA patient cohort and tested on an independent patient dataset (GSE13041). Results reflect classification accuracy of testing incremental combinations of subnetworks (e.g. classification results of Subnetwork 3 represent the use of subnetworks 1, 2, and 3 in classifying the independent patient dataset). (PDF) [file pcbi.1003237.s006.pdf]

Table S2. Classification results of CRANE subnetworks discovered from the TCGA patient cohort and tested on an independent patient dataset (GSE13041). Results reflect classification accuracy of testing incremental combinations of subnetworks (e.g. classification results of Subnetwork 3 represent the use of subnetworks 1, 2, *and* 3 in classifying the independent patient dataset).

| Number of Subnetwork 1 | Short Survival(True) | Long Survival(True) | Total |
|---|---|---|---|
| Short Survival(CRANE) | 32 | 24 | 56 |
| Long Survival(CRANE) | 9 | 26 | 35 |
| Total | 41 | 50 | 64% |

| Number of Subnetwork 2 | Short Survival(True) | Long Survival(True) | Total |
|---|---|---|---|
| Short Survival(CRANE) | 33 | 18 | 51 |
| Long Survival(CRANE) | 8 | 32 | 40 |
| Total | 41 | 50 | 71% |

| Number of Subnetwork 3 | Short Survival(True) | Long Survival(True) | Total |
|---|---|---|---|
| Short Survival(CRANE) | 31 | 19 | 50 |
| Long Survival(CRANE) | 10 | 31 | 41 |
| Total | 41 | 50 | 68% |

| Number of Subnetwork 4 | Short Survival(True) | Long Survival(True) | Total |
|---|---|---|---|
| Short Survival(CRANE) | 35 | 16 | 51 |
| Long Survival(CRANE) | 6 | 34 | 40 |
| Total | 41 | 50 | 76% |

| Number of Subnetwork 5 | Short Survival(True) | Long Survival(True) | Total |
|---|---|---|---|
| Short Survival(CRANE) | 26 | 3 | 29 |
| Long Survival(CRANE) | 15 | 47 | 62 |
| Total | 41 | 50 | 80% |
